# An m5C methylation regulator-associated signature predicts prognosis and therapy response in pancreatic cancer

**DOI:** 10.3389/fcell.2022.975684

**Published:** 2022-08-19

**Authors:** Duo Yun, Zhirong Yang, Shuman Zhang, Hai Yang, Dongxue Liu, Robert Grützmann, Christian Pilarsky, Nathalie Britzen-Laurent

**Affiliations:** ^1^ Division of Surgical Research, Department of Surgery, Universitätsklinikum Erlangen, Friedrich-Alexander-Universität Erlangen-Nürnberg, Erlangen, Germany; ^2^ Department of Surgery, Universitätsklinikum Erlangen, Friedrich-Alexander-Universität Erlangen-Nürnberg, Erlangen, Germany

**Keywords:** 5-methylcytosine (m5C), pancreatic cancer, prognosis, targeted drug therapy, tumor microenvironment (TME), immune cells infiltration

## Abstract

Pancreatic ductal adenocarcinoma (PDAC) is the most aggressive digestive malignancy due to frequent late-stage diagnosis, rapid progression and resistance to therapy. With increasing PDAC incidence worldwide, there is an urgent need for new prognostic biomarkers and therapy targets. Recently, RNA methylation has emerged as a new tumorigenic mechanism in different cancers. 5-methylcytosine (m5C) is one of the most frequent RNA modifications and occurs on a variety of RNA species including mRNA, thereby regulating gene expression. Here we investigated the prognostic role of m5C-regulator-associated transcriptional signature in PDAC. We evaluated m5C-regulator status and expression in 239 PDAC samples from publicly available datasets. We used unsupervised consensus clustering analyses to classify PDACs based on m5C-regulator expression. From the resulting signature of differentially expressed genes (DEGs), we selected prognosis-relevant DEGs to stratify patients and build a scoring signature (m5C-score) through LASSO and multivariate Cox regression analyses. The m5C-score represented a highly significant independent prognostic marker. A high m5C-score correlated with poor prognosis in different PDAC cohorts, and was associated with the squamous/basal subtype as well as activated cancer-related pathways including Ras, MAPK and PI3K pathways. Furthermore, the m5C-score correlated with sensitivity to pathway-specific inhibitors of PARP, EGFR, AKT, HER2 and mTOR. Tumors with high m5C-score were characterized by overall immune exclusion, low CD8^+^ T-cell infiltration, and higher PD-L1 expression. Overall, the m5C-score represented a robust predictor of prognosis and therapy response in PDAC, which was associated with unfavorable molecular subtypes and immune microenvironment.

## Introduction

Pancreatic ductal adenocarcinoma (PDAC) is one of the deadliest human malignant tumors, with an overall 5-year survival rate below 11%. It represents the seventh leading cause of cancer death globally for both sexes. In high-development-index countries, its mortality rank is estimated to increase from fifth to second or third by 2030 (Rahib et al., 2014; Siegel et al., 2022). The high mortality of PDAC has been notably imputed to the absence of obvious symptom at early stages, resulting in the majority of patients being diagnosed with locally advanced or metastatic tumors (Edwards et al., 2005; Arslan et al., 2010). While late diagnosis reduces the number of treatment options, PDAC is also highly recalcitrant to most therapies, including immunotherapy (Sung et al., 2021). Thus, it is crucial to develop new prognostic and predictive tools to improve therapeutic intervention in a personalized manner.

Post-transcriptional RNA methylation has emerged as an important epigenetic regulatory mechanism, which has recently gathered increasing interest, notably for its association with human cancer progression (Haruehanroengra et al., 2020; Han et al., 2021). 5-Methylcytosine (m5C) is one of the most prevalent RNA modifications, affecting mRNAs, tRNAs and rRNAs (Chen et al., 2021). The methylation of RNA cytosines is reversibly and specifically modulated by regulators including methyltransferases (writers) and demethylases (erasers), and is specifically recognized by binding proteins (readers) (Bohnsack et al., 2019). The m5C-modification of mRNAs has been shown to regulate their splicing, transport, translation, stabilization or degradation (Boo and Kim, 2020; Chen et al., 2021). Dysregulation of m5C-regulator gene expression is involved in pathophysiological processes such as cell proliferation, cell death or immune modulation, and has been observed in various cancers (Zhang et al., 2021a; Xu et al., 2021b). Changes in m5C regulators expression or m5C-regulator-associated signatures have been found to correlate with prognosis, immune infiltration and/or therapy response in different solid tumor entities (Zhang et al., 2021b; Wood et al., 2021).

The purpose of the present study was to investigate the prognostic relevance of tumor-associated dysregulation of the m5C-modification pathway for pancreas cancer patients. We combined genomic, transcriptional and clinical data from 239 PDAC patients to test the prognostic power of m5C-regulators and m5C-associated transcriptional signatures. We investigated the impact of different m5C-associated patterns on survival, gene/pathway expression, molecular subtypes, therapy response and immune infiltration in order to provide a comprehensive evaluation of the pathophysiological consequences of the m5C pathway dysregulation in PDAC.

## Material and methods

### Collection and pre-processing of pancreatic ductal adenocarcinoma gene expression public datasets

The gene and clinical information data were searched from the databases of Gene Expression-Omnibus (GEO) (https://www.ncbi.nlm.nih.gov/geo/), the Cancer Genome Atlas (TCGA) (https://portal.gdc.cancer.gov/) and the supplementary data of Bailey’s cohort (https://www.nature.com/articles/nature16965#Sec11). For TCGA datasets, the fragments per kilobase of transcript per million mapped reads (FPKM) values were transformed into transcripts per kilobase million (TPM) values by using the R package “limma” (v. 3.50.3). For building and internal testing of prognostic model, we combined TCGA PAAD cohort and GSE57495 (RMA value) cohort data (*n* = 239). Batch effects were corrected using the “ComBat” function (batchType, par.prior = TRUE) of R package “sva” (v. 3.42.0) (Leek et al., 2012). Principal components analysis (PCA) algorithm was used to confirm the results of the batch effects correction based on the normalized sequencing data using the “prcomp” (scale. = TRUE) function in R package “limma” (v. 3.50.3). For external validation of the prognostic model, data from the GSE21501(data were Lowess-normalized and then log2 ratio was taken) cohort and Bailey’s cohort (normalized RSEM data were converted to counts per million (c.p.m.) and log2 transformed) were used independently. For comparison with other molecular classifications, Moffitt’s cohort data (GSE71729: data were non-negative normalized log2 Cy5 signal), Bailey’s cohort data, Collision’s cohort data (GSE17891; RMA log2 data) were used. The copy number variation data of PDAC patients were downloaded from the UCSC Xena database (https://xena.ucsc.edu/) and the somatic mutation data were downloaded from the TCGA database. The genetic landscape of 12 m5C-regulators in 23 pairs of chromosomes and the variation were visualized using the R-package “Rcircos” (v. 1.2.2) (Zhang et al., 2013). The mutation status was visualized using the R-package “maftools” (v. 2.10.5) (Mayakonda et al., 2018). The expression of m5C regulators in pancreatic tumors and normal tissues was analyzed with the online tools GEPIA2 (http://gepia2.cancer-pku.cn/#analysis) (Tang et al., 2019) using data from TCGA and GTEx (https://gtexportal.org/home/) (Carithers and Moore, 2015). Patients without survival data were removed from further survival-related analyses. The cell line RNA-seq data were obtained from the Moffitt’s cohort data (GSE71729) and GSE165949 (Alfarano et al., 2022), the latter as data normalized by “summarizeOverlaps” from the R package GenomicRanges (v.1.38). Basic information of these datasets is listed in Supplementary Table S1 **,** and the study workflow is presented in Supplementary Figure S1.

### Unsupervised consensus cluster analysis

The “Consensusclusterplus” function (maxK = 9, reps = 50, clusterAlg = “pam”, distance = “euclidean”) was used to perform unsupervised consensus clustering analysis in order to classify PDAC patients based on normalized RNA sequencing data in R package “Consensusclusterplus” (v. 1.58.0) (Wilkerson and Hayes, 2010). The best k-value was selected based on the clustering effect and the cumulative distribution function (CDF). The result of classification was confirmed by Principal Component Analysis (PCA) based on the normalized sequencing data using the “prcomp” (scale. = TRUE) function in R basic package.

### Gene set variation analysis

The “gsva” function (geneSets, min.sz = 10, max.sz = 500,) was used to analyze the transcriptome gene enrichment (Hänzelmann et al., 2013) using the gene document named “c2.cp.biocarta.v7.4.symbols.gmt” from the MSigDB database (https://www.gsea-msigdb.org). We used the R-packages of “gene set variation analysis (GSVA)” (v. 1.42.0) “limma” (v. 3.50.3), “GSEABase” (v. 1.56.0) to analyze the different pathways between the different m5C-clusters.

### Construction and validation of the m5C-related prognostic risk scoring signature (m5C-score)

The differentially expression genes (DEGs) between three m5C-clusters were analyzed by using “lmFit”, “contrasts.fit” and “eBayes” in the R package “limma” (v.3.50.3) with a *p*-value filter adjusted to 0.001. We determined the differentially expressed genes (DEGs) between m5C-cluster A and B, A and C, or B and C. The potential functions of these DEGs were then analyzed by Gene Ontology (GO) annotation and Kyoto Encyclopedia of Genes and Genomes (KEGG) enrichment pathway analysis by using the “enrichKEGG” (organism = “hsa”, pvalueCutoff = 1, qvalueCutoff = 1) and “enrichGO” (OrgDb = org.Hs.eg.db, pvalueCutoff = 1, qvalueCutoff = 1) function in the R-package “clusterProfiler” (v. 4.2.2) and “org.Hs.eg.db”(version 3.14.0). An FDR < 0.05 was considered as significant. To build the m5C-score, we first randomly attributed the 239 patients to a training set (120 patients) and a testing set (119 patients). Univariate Cox regression analysis was applied on the training set to screen prognostic related DEGs with a *p-*value < 0.05 by using the function “coxph” in the R-package “survival” (v. 3.3.1). Then the least absolute shrinkage and selection operator (LASSO) regression analysis was used to avoid over-fitting by using the “glmnet” function (family = “cox”, maxit = 1000) function in the R-package “glmnet” (v. 4.1.4) where the value of the lambda is chosen by the smallest likelihood deviance. Finally, multivariate Cox regression analysis was used to build the multivariate Cox proportional hazards regression model by using the “coxph” function in the R package “survival” (v. 3.3.1). The resulting m5C-score was constructed by four genes differentially regulated between clusters A, B and C (HIPK3, ZFAND4, DPP8, HIPK2). The signature could be represented using the formula: m5C-score = $\Sigma_{n}^{i}\left(Coefi*Xi\right)$ , in which *X* represents the expression level of each m5C-score gene and Coef represents the coefficient of each m5C-score gene in the multivariate Cox proportional hazards regression model. The m5C-score was then validated with the testing set, as well as two independent datasets (GSE21501 and Bailey cohort).

### Gene set enrichment analysis

The gene set enrichment analysis (GSEA) software (version 4.1.0) was used to determine which pathways were significantly different between high- and low-m5C-score groups using the GSEA software (https://www.broadinstitute.org/gsea/). The KEGG-database “c2.cp.kegg.v7.5.symbols.gmt” was chosen as the reference database. The number of random permutations was set to 1000. The gene expression data in the TCGA-PAAD database were grouped via the m5C-score and recorded as the high- and low-m5C-score groups. The phenotype labels were set as high-m5C-score group versus low-m5C-score group patients. Collapse/Remap to gene symbols was set as “No_Collapse”. The other parameters were the default parameters in the GSEA software. A pathway with FDR q < 0.05 was defined as statistically significant.

### Target drugs sensitivity prediction

The sensitivity to target drugs was determined by estimating the half-maximal inhibitory concentration (IC50) with the “pRRopheticPredict” (selection = 1) function in the R-package “pRRophetic” (version 0.5) (Geeleher et al., 2014). Common targets (Liu et al., 2021) being used in clinic or clinical trials were analyzed.

### Immune cells infiltration and exclusion analyses

Cell type Identification By Estimating Relative Subsets Of RNA Transcripts (CIBERSORT) (Newman et al., 2015) (https://cibersort.stanford.edu) was used to quantify the proportions and distributions of tumor-infiltrating immune cells (TIICs) based on the RNA-seq data. The single-sample gene set enrichment analysis (ssGSEA) (Barbie et al., 2009) method was used to calculate the relative enrichment of immune cells for each PDAC patient by using the “gsva” (method = “ssgsea”, kcdf = “Gaussian”, abs.ranking = TRUE) function in the R-package “GSVA” (v. 1.42.0). The marker genes for each infiltrating immune cell type were based on the results of Charoentong and colleagues (Charoentong et al., 2017). The Tumor Immune Dysfunction and Exclusion (TIDE) (http://tide.dfci.harvard.edu/) algorithm was used to predict PDAC immune exclusion of samples from the TCGA-PAAD cohort (Jiang et al., 2018).

### Statistical analysis

For data analyses, we used the R-Studio (4.1.2) suite. For the comparison of data between two groups, the Student’s *t*-test was used for analyzing the quantitative statistics of normally distributed variables and the Wilcoxon rank sum test was used for analyzing the quantitative statistics of non-normally distributed variables. If the comparisons were between more than two groups, one-way ANOVA was used for parametric methods and the Kruskal–Wallis test was used for the non-parametric methods. The Kaplan–Meier (K-M) method and log-rank test were used for survival analyses. The fulfillment of the proportional hazards assumption was verified for all survival analyses with a significant survival difference (log-rank test *p* < 0.05) by hierarchical regression using the SPSS software. The “surv_cutpoint” function was used to identified the best cutoff in the R-package “survminer” (v. 0.4.9) for the K-M analyses of single genes and the external validation of the m5C-score. The specificity and sensitivity of factors were analyzed by receiver operating characteristic (ROC) curve, and the area under the curve (AUC) were calculated with the “timeROC” (weighting = “aalen”) function in the R-package “timeROC” (v.0.4). The degree of correlation of the m5C-score with gene expression and immune infiltration was determined by Spearman analyses. A *p*-value < 0.05 was regarded as statistically significant.

## Results

### Genetic variation and prognostic value of m5C-regulator expression in pancreatic ductal adenocarcinoma

**FIGURE 1:**
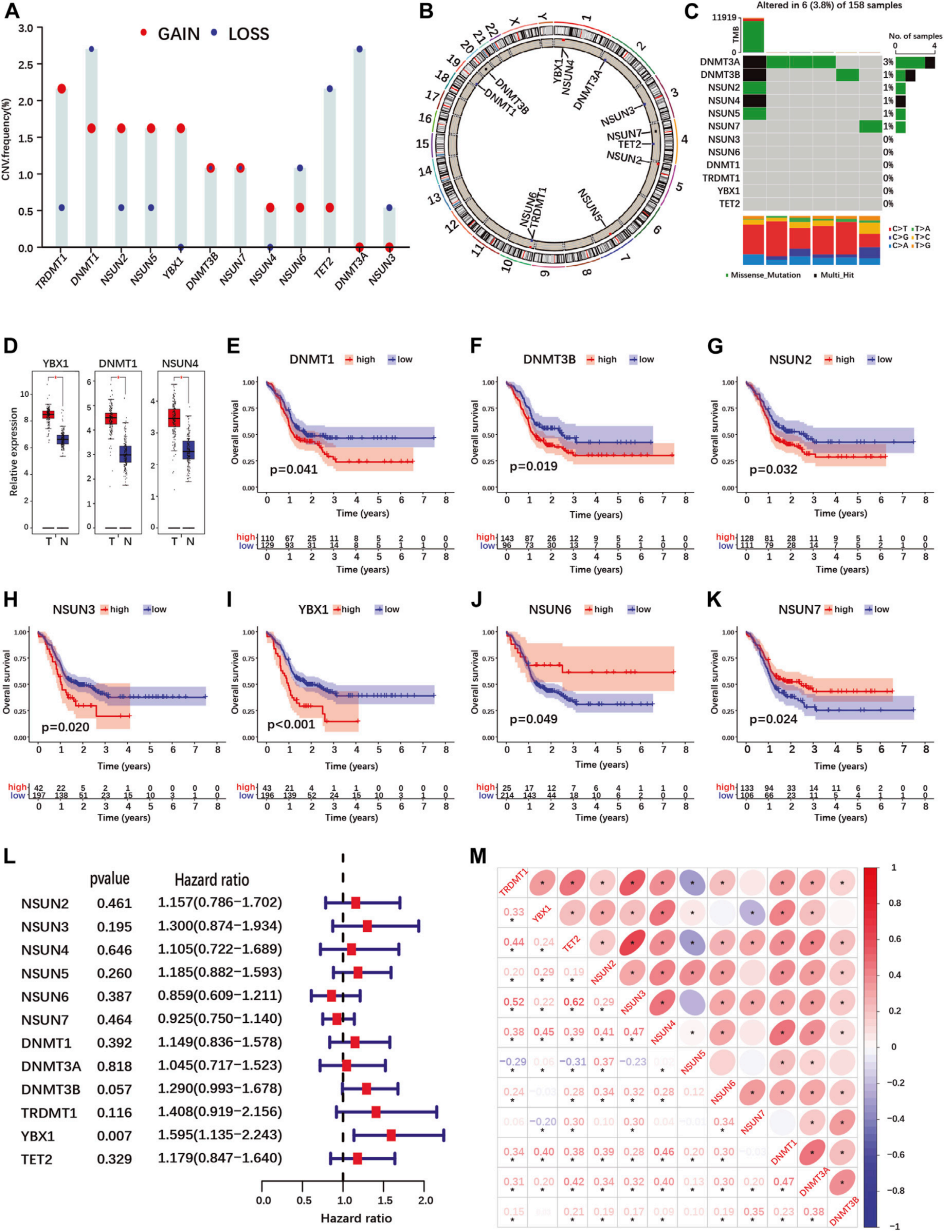
Landscape of genetic variation, differential expression, prognosis and correlations analyses of m5C-regulator genes in PDAC. **(A,B)** Copy number variation (CNV) frequency **(A)** and location on chromosomes **(B)** of m5C-regulator genes in the TCGA-PAAD cohort. Red dots represent amplifications, blue dots deletions and black dots equal amplification and deletion frequency. **(C)** The somatic mutation status of m5C- regulator genes in the TCGA-PAAD cohort. The upper bar represents tumor mutation burden (TMB), the right bar the number of PDAC patient with a mutation in one or more m5C-regulator gene, and the bar below the distribution of conversions in each sample. **(D)** Differential expression of m5C-methylation regulators between PDAC and normal pancreas tissue in the TCGA-PAAD and GTEx cohorts. Tumor, red; Normal, blue. **(E–K)** K-M curves illustrating OS between high- and low- expression of m5C-regulator genes analyzed by log-rank test in the TCGA-PAAD and GSE57495 cohorts. **(L)** Univariate Cox regression analyses of OS of m5C-regulator genes in the TCGA-PAAD and GSE57495 cohorts. **(M)** Correlation analyses between m5C-regulator gene expression by Spearman analyses. *p-value < 0.05.

Twelve known m5C-methylation regulators including ten writers, one eraser and one reader were selected for analysis (Supplementary Table S2, Supplementary Figure S1) (Ghanbarian et al., 2016; Bohnsack et al., 2019; Chen et al., 2019; DeNizio et al., 2019). Copy number variations (CNV) and point mutations of m5C-regulator genes were rare in the TCGA-PAAD cohort with a frequency below 3 and 4%, respectively (Figures 1A–C). In addition, the presence of mutations in m5C-regulator genes was not associated with the survival of PDAC patients (Supplementary Figure S2A).

Using data from the TCGA-PAAD and GTEx cohorts, we compared m5C-regulator expression between tumor and normal tissues. Only three genes (YBX1, DNMT1 and NSUN4) out of twelve were significantly differentially expressed in tumors, all showing an up-regulation (Figure 1D; Supplementary Figure S2G). We then assessed the prognostic value of m5C-methylation regulators expression for PDAC patients in our cohort (TCGA-PAAD + GSE57495). Seven out of twelve m5C-methylation regulators showed a significant correlation between gene expression and overall patient survival (Figures 1E–K and Supplementary Figures S2B–F). High expression of DNMT1, DNMT3B, NSUN2, NSUN3 and YBX1 was associated with a worse prognosis (Figures 1E–I). On the contrary high expression of NSUN6 and NSUN7 correlated with a better prognosis (Figures 1J,K). However, only one gene, the m5C-reader YBX1, was significantly associated with PDAC prognosis in the Univariate Cox regression analysis (Figure 1L).

Given that the expression of several m5C-methylation regulators correlated positively in PDAC (Figure 1M), we investigated whether pancreatic cancers could be classified based on m5C-regulator gene expression. Unsupervised consensus clustering identified 3 subgroups named m5C-clusters A, B and C in our PDAC cohort (Supplementary Figures S3A–E; Supplementary Table S3). GSVA pathway analysis revealed differentially regulated cancer pathways between the three m5C-clusters, including KRAS-related pathways (MAPK-, mTOR-, EGF- and ERK-pathways), cell death pathways (TNFR1-, FAS- and death-pathways), and cancer-related pathways (Wnt-, Gleevec-, MET and CREB-pathways) (Supplementary Figure S3F). The expression of each m5C-regulator was also significantly different between three m5C-clusters (Supplementary Figure S3G). However, the different clusters did not correlate with the survival of PDAC patients in our cohort (Supplementary Figure S3H).

### Identification of prognosis-relevant m5C-regulated gene subtypes

**FIGURE 2:**
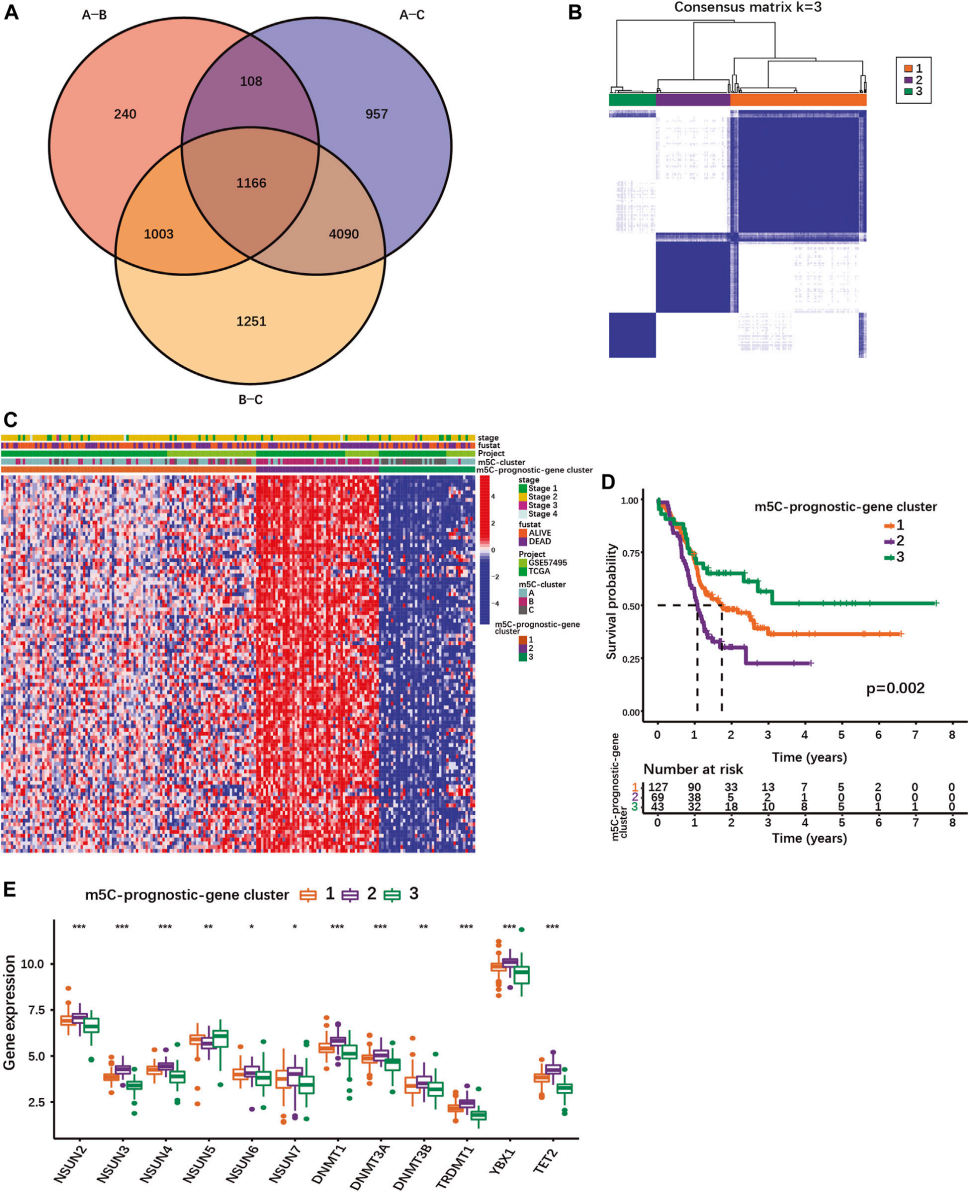
Identification of different PDAC subgroups based on m5C-related prognostic DEGs. **(A)** Venn diagram showing 1166 DEGs between different m5C-clusters. **(B)** Consensus matrix for optimal k = 3 clusters using the TCGA-PAAD and GSE57495 cohorts analyzed by unsupervised consensus clustering analyses based on the 1166 m5C-related prognostic DEGs. **(C)** Heatmap showing the expression of m5C-related prognostic DEGs with survival status, stage, m5C-clusters, m5C-prognostic-gene clusters. Columns represented patients and rows m5C-related prognostic DEGs. **(D)** The K-M curves show OS for m5C-prognostic-gene clusters 1/2/3 analyzed by log-rank test in the TCGA-PAAD and GSE57495 cohorts. **(E)** Expression of m5C-regulators in the three m5C-prognostic-gene clusters; *p < 0.05; **p < 0.01; ***p < 0.001.

Since patient clustering according to m5C-regulator expression could not predict survival but was associated with transcriptional changes affecting several pancreatic cancer pathways, we investigated whether PDAC samples could be clustered according to m5C-related gene expression. In total, 1166 genes were differentially expressed between all three m5C-clusters (Figure 2A; Supplementary Table S4-4). Both KEGG and GO enrichment analyses showed that m5C-related differentially expressed genes (DEGs) were enriched in pathways regulating RNA regulation and processing (Supplementary Figures S4A,B; Supplementary Table S5-1/2), and in cancer-related pathways (Supplementary Figures S4A,B; Supplementary Table S5-1/2). This indicated that the changes in m5C-regulator expression in cancer have functional consequences impacting RNA regulation and cancer-pathway gene expression.

Using univariate Cox regression analysis, 100 m5C-related DEGs were identified as statistically significant prognostic markers in PDAC (Supplementary Table S6). Based on their expression, PDAC patients of our cohort could be stratified into 3 subgroups named m5C-prognostic-gene cluster 1, 2 and 3 (Figures 2B,C; Supplementary Figures S4C–E). The m5C-prognostic-gene cluster 3 was characterized by an overall low expression of the prognostic DEGs, and the highest overall survival rate (Figures 2C,D). The m5C-prognostic-gene cluster 2 was associated with a high expression of the prognostic DEGs and the lowest survival rate, while the m5C-prognostic-gene cluster 1 exhibited a mixed phenotype with intermediate survival rate (Figures 2C,D). Of note, the expression of each m5C-regulator was also significantly different between the three m5C-prognostic-gene clusters (Figure 2E). Except for NSUN5, all m5C-regulator genes were up-regulated in cluster 2 (Figure 2E), suggesting that the overexpression of m5C-regulators induces the expression of multiple genes involved in tumorigenesis.

### Construction and validation of a prognostic model based on an m5C-related gene expression signature (m5C-score)

Next, we build a scoring signature (m5C-score) based on the 100 m5C-related prognostic DEGs to be used as prognostic model for PDAC patients. First, the patients of our cohort were randomly distributed into a training set and a testing set (Supplementary Table S7). LASSO regression analysis was performed on the training set using the m5C-related prognostic genes to avoid over-fitting (Supplementary Figures S5A,B). The multivariate Cox proportional hazards regression model was used to obtain the scoring signature (m5C-score), which is composed of four prognostic DEGs: Homeodomain Interacting Protein Kinase 3 (HIPK3), Zinc Finger AN1-Type Containing 4 (ZFAND4), Dipeptidyl Peptidase 8 (DPP8) and Homeodomain Interacting Protein Kinase 2 (HIPK2). The correlation coefficients of each gene was computed by multivariate Cox regression analysis and the m5C-score was calculated as follows: m5C-score = (1.0722 × exp[HIPK3]) + (−1.168 × exp[ZFAND4]) + (0.9293 × exp[DPP8]) + (−0.6607 × exp[HIPK2]), where “exp” means the expression of each m5C-score gene. Positive and negative coefficients indicated positive or negative correlation between single gene expression and the m5C-score, respectively.

**FIGURE 3:**
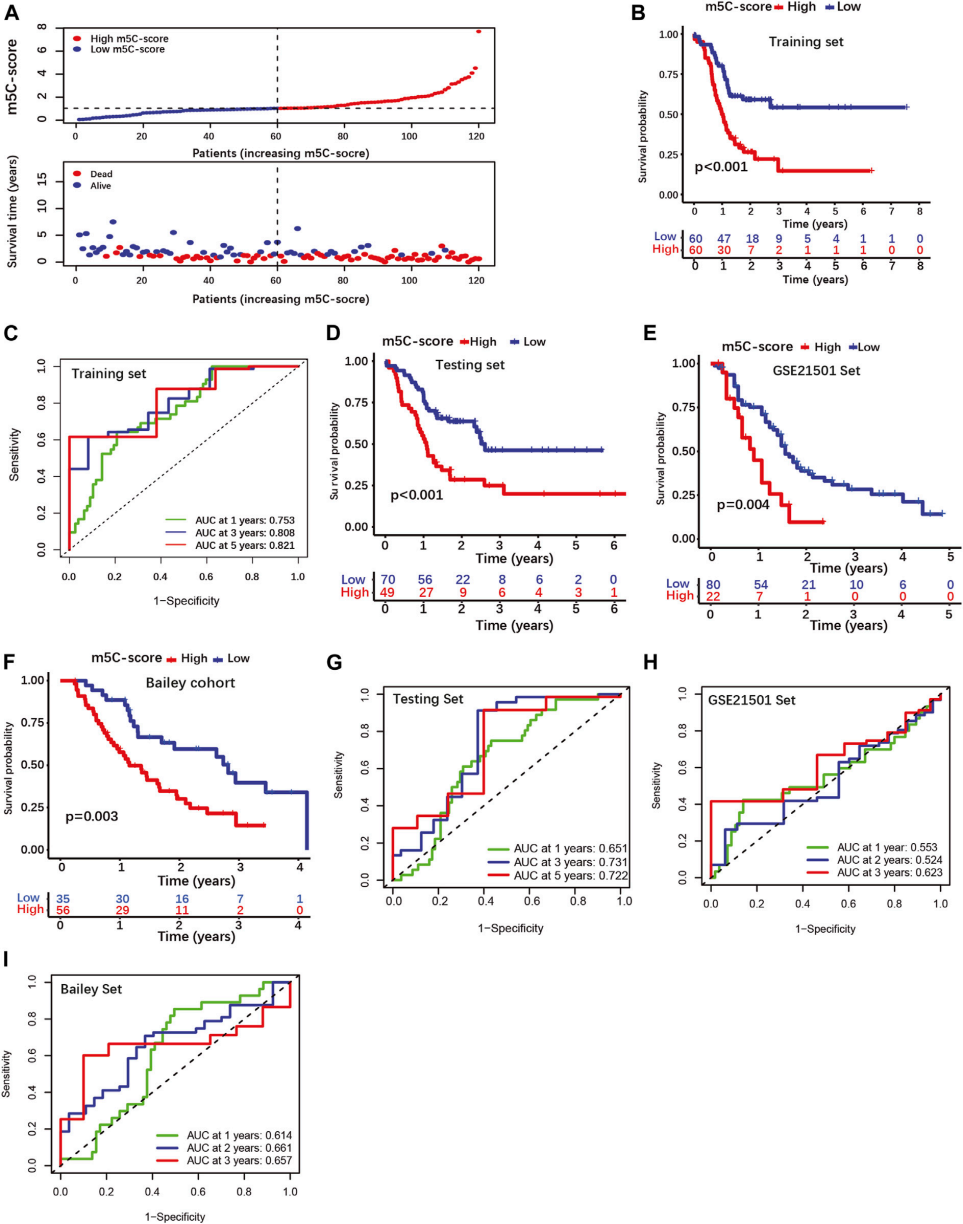
Construction and validation of a four-gene scoring signature (m5C-score) prognostic model. **(A)** Distributions of PDAC patients and survival status according to m5C-score in the training set. **(B)** K-M curves illustrating OS according to the m5C-score analyzed by log-rank test in the training set. **(C)** ROC curves for m5C-score prediction of OS at 1, 3 and 5 years in the training set. **(D–F)** K-M curves illustrating OS according to the m5C-score analyzed by log-rank test in the testing set and external cohorts (GSE21501 set, Bailey cohorts). **(G–I)** ROC curves of m5C-score prediction of OS at 1, 3 and 5 years in the testing and external cohorts.

Then, patients were classified into high-m5C-score and low-m5C-score groups using the median value of the m5C-score as cut-off (Figure 3A; Supplementary Table S8-1). Patients with low m5C-score survived significantly longer than patients with high m5C-score (Figure 3B). The area under the curve (AUC) of time-dependent receiver operating characteristic (ROC) curve was 0.821 for the 5-year overall survival (OS), showing that the m5C-score is an excellent predictor of survival (Figure 3C). The prognostic value of the m5C-score was then validated in the testing set and in two independent cohorts (GSE21501, Bailey cohort). Here again, patients with low m5C-score showed significantly longer survival than patients with high m5C-score, and the m5C-score could predict survival with good accuracy (Figures 3D–I; Supplementary Tables S8-2/3/4).

**FIGURE 4:**
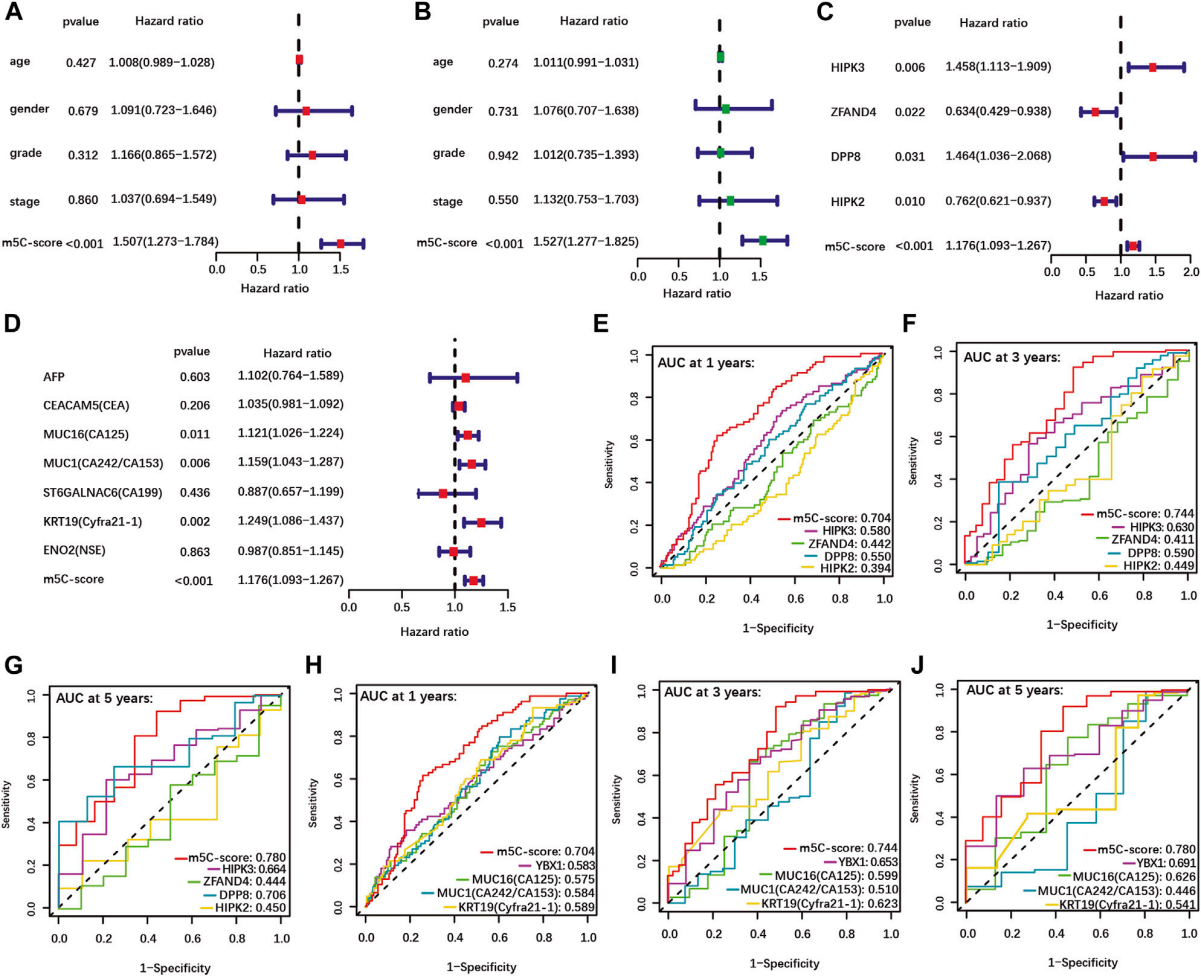
Higher predictive value of the m5C-score compared with clinical factors, individual m5C-regulator genes and tumor marker genes. **(A–B)** Univariate **(A)** and multivariate **(B)** Cox regression analyses of OS of m5C-score and clinical factors from the TCGA-PAAD cohort. **(C–D)** Univariate Cox regression analyses of OS of m5C-score and individual m5C-score genes **(C)** and tumor marker genes **(D)** in the TCGA-PAAD and GSE57495 cohorts. **(E–J)** The AUC of ROC curves shows the predictive value of OS at 1, 3 and 5 years of m5C-score compared to individual m5C-score genes **(E–G)**, or the prognostic-related m5C-regulator YBX1 and serum tumor marker genes **(H–J)**.

Univariate and multivariate Cox regression analyses performed using clinical data available for the TCGA-PAAD cohort showed that the m5C-score is an independent risk factor for PDAC patients, while age, gender, tumor grade and stage were not significant (Figures 4A,B). In addition, each individual m5C-score gene and common PDAC serum tumor markers were analyzed with the m5C-score by univariate Cox regression analysis in our cohort (Figures 4C,D). Each m5C-score gene taken individually (Figure 4C; Supplementary Figures S5C–F) and the marker genes MUC16, MUC1 and KRT19 (Figure 4D) were significantly associated with prognosis. All the statistically significant parameters (*p* < 0.05) were then selected to draw ROC curves, which showed that the m5C-score had the highest AUC at 1-, 3- and 5-year OS compared with either the m5C-score individual genes (Figures 4E–G), the m5C-methylation reader YBX1 or common PDAC serum tumor markers (Figures 4H–J). Overall, these results established the m5C-score is a robust predictive tool for survival of PDAC patients, that was superior to m5C-regulators or common tumor markers.

### A high m5C-score is associated with mutation and transcriptomic regulation of pancreatic cancer-related genes

**FIGURE 5:**
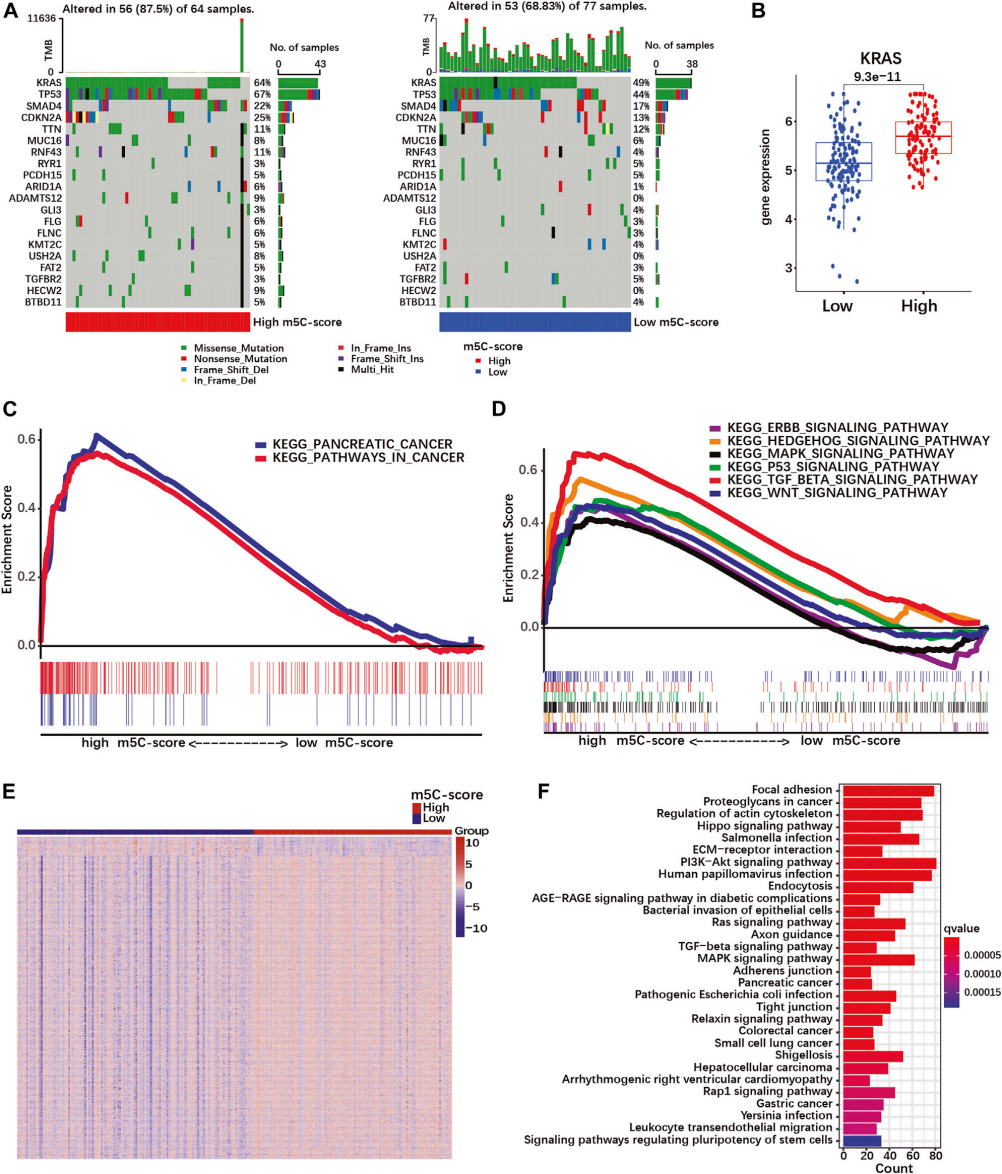
A high m5C-score is associated with increased mutation rate and expression of PDAC-related genes and pathways. **(A)** Somatic mutation status in high- and low-m5C-score groups in the TCGA-PAAD cohort. The upper bar represents tumor mutation burden (TMB), the right bar the number of PDAC patient with mutation for each of the 20 most frequently mutated genes in PDAC, and the bar below high- and low-m5C-score group patients. **(B)** KRAS expression in high- and low-m5C-score tumors from the TCGA-PAAD + GSE57495 cohort. **(C,D)** Gene set enrichment analysis (GSEA) between high- and low-m5C-score groups in the TCGA-PAAD cohort. **(E)** Heatmap of DEGs between high- and low-m5C-score groups. Columns represent patients and rows DEGs between high- and low-m5C-score groups. **(F)** KEGG pathway analyses for genes differentially expressed between high- and low-m5C-score PDACs.

To further characterize the pathobiological significance of the m5C-score, somatic mutations were compared in PDACs with high and low m5C-score. The frequency of somatic mutations in the TCGA cohort was higher in the high-m5C-score group (87.5%) than in the low-m5C-score group (68.83%, Figure 5A). In particular, KRAS, TP53, SMAD4 and CDKN2A were more frequently mutated in the high-m5C-score group. In agreement with these results, a higher expression of KRAS was observed in tumors with high-m5C-score compared to low-m5C-score (Figure 5B).

Then, GSEA pathway analysis was performed to investigate whether specific pathways could be related to the m5C-score. PDAC-related pathways were enriched in the high-m5C-score group, which included “pathways in cancer”, “pancreatic cancer”, and several signaling pathways (TGF-beta, MAPK, p53, hedgehog, ERBB and Wnt; Supplementary Table S9; Figures 5C,D). In addition, the genes differentially expressed between high-m5C-score and low-m5C-score groups (Figure 5E; Supplementary Table S10) were enriched in many malignancy-related pathways (Figure 5F; Supplementary Table S11). Taken together, these results showed an association between a high-m5C-score, mutation rates and transcriptional regulation of pancreatic cancer-related pathways, including the MAPK pathway doenstream of KRAS, and the TGFβ pathway.

### A high m5C-score is associated with the squamous/basal pancreatic ductal adenocarcinoma subtype

**FIGURE 6:**
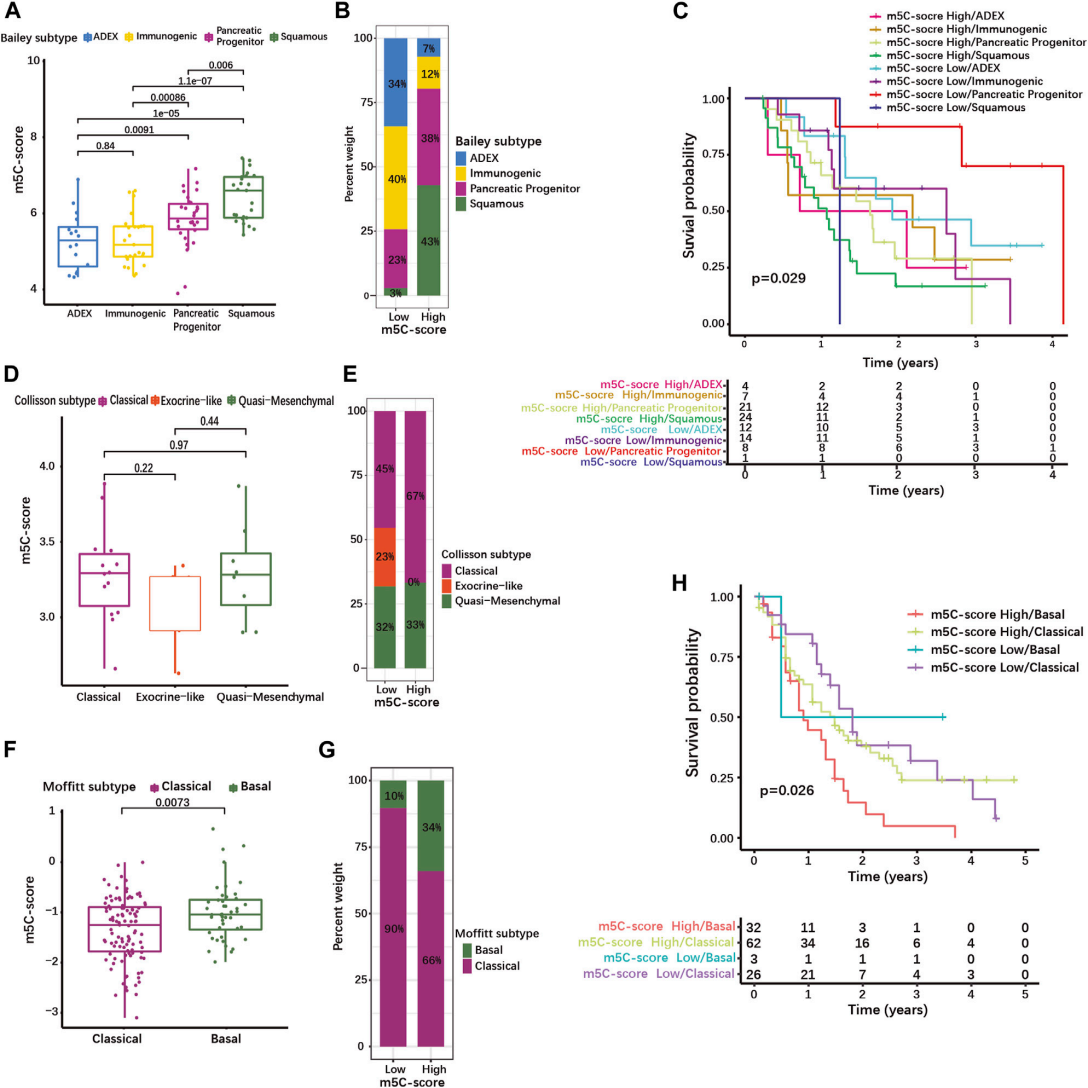
High m5C-score is associated with the squamous/basal molecular subtype and poor prognosis in PDAC. **(A)** m5C-score levels in the different molecular subtypes defined by Bailey et al.; **(B)** Distribution of Bailey’s molecular subtypes in high- and low-m5C-score PDACs; **(C)** K-M curves illustrating OS according to the combination of the m5C-score with Bailey’s molecular subtypes analyzed by log-rank test. **(D)** m5C-score levels in the different molecular subtypes defined by Collisson et al.; **(E)** Distribution of Collisson’s molecular subtypes in high- and low-m5C-score PDACs. **(F)** m5C-score levels in the two molecular subtypes defined by Moffitt et al.; **(G)** Distribution of Moffitt’s molecular subtypes between high- and low-m5C-score PDACs; **(H)** K-M curves illustrating OS according to the combination of the m5C-score with Moffitt’s molecular subtypes analyzed by log-rank test.

We then evaluated whether the m5C-score correlated with the common molecular subtypes of PDAC. For this, we used classifications published by Bailey, Collisson, and Moffitt et al. (Collisson et al., 2011; Moffitt et al., 2015; Bailey et al., 2016). The Bailey classification distinguishes 4 molecular subtypes (Bailey et al., 2016). The Squamous subtype showed a significantly higher m5C-score compared to the Progenitor, the Immunogenic and the Aberrantly Differentiated Endocrine Exocrine (ADEX) subtypes (Figure 6A, Supplementary Table S8-4). The proportion of tumor of the Squamous and Progenitor subtypes was higher in the high-m5C-score group, while Immunogenic and ADEX subtypes were over-represented in the low-m5C-score group (Figure 6B). Survival analysis revealed that patients with high-m5C-score combined with Squamous subtype had the worst prognosis, while patients with low-m5C-score combined with Progenitor subtype showed the best prognosis (Figure 6C).

The Collison classification differentiates Classical, Exocrine-like and Quasi-mesenchymal subtypes (Supplementary Table S8-5) (Collisson et al., 2011). The m5C-score in the Exocrine-like subgroup was marginally lower than in the other two subtypes (Figure 6D), and the proportion of Exocrine-like tumors was drastically reduced in the high-m5C-score group (Figure 6E).

In the Moffit cohort (Moffitt et al., 2015), tumors are classified as either Basal-like or Classical (Supplementary Table S8-6). The Basal-like subtype was associated with a higher m5C-score (Figure 6F) and the high-m5C-score group displayed a higher proportion of Basal-like tumors (Figure 6G). In the Moffitt cohort, Basal-like tumors with high-m5C-score had the worst prognosis, while low-m5C-score combined with Classical subtype showed the best prognosis (Figure 6H). A strong overlap has been described between the Classical/Pancreatic Progenitor subtypes on one side, and the Squamous/Basal-like/Quasi-mesenchymal subtypes associated with poor a prognosis on the other side (Xu Z. et al., 2021). Taken together, this means that a high m5C-score is strongly associated with the Squamous/Basal-like subtype, which represents a more aggressive phenotype with a poor prognosis.

### The m5C-score correlates with targeted therapy response in pancreatic ductal adenocarcinoma

Targeted therapy of PDAC remains limited, with the EGFR-inhibitor Erlotinib being the only approved drug for treatment of advanced inoperable PDAC in combination with gemcitabine. However, multiple drugs targeting the KRAS signaling pathway, either upstream or downstream, as well as DNA repair are being tested in preclinical and clinical trials (Liu et al., 2021). We used the R package “pRRophetic” to predict the sensitivity of tumors with high or low m5C-score towards drugs targeting these pathways.

**FIGURE 7:**
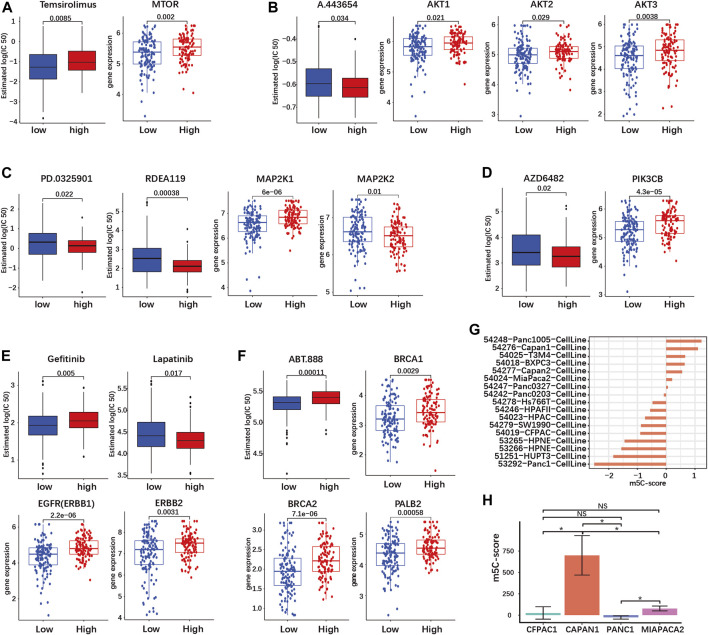
The m5C-score can predict target drug response in PDAC. Target drug IC50 prediction and differential expression analyses of target genes between high- and low-m5C-score groups in the TCGA-PAAD and GSE57495 cohorts are given for **(A)** mTOR inhibitor (MTOR gene); **(B)** AKT inhibitor (AKT 1/2/3 genes); **(C)** MEK1 and 2 inhibitors (MEK1 and MEK2 genes - MAP2K1/2); **(D)** PI3K inhibitor (PIK3CB gene); **(E)** EGFR and HER2 inhibitors (EGFR—ERBB1—and HER2—ERBB2—genes; **(F)** PARP inhibitor (BRC1/2 and PALB2 genes). **(G)** The m5C-score comparison of different PDAC cell line type from GSE165949. **(H)** The m5C-score comparison of different PDAC cell lines from the Moffitt’s cohort.

First, we investigated drugs targeting the signaling downstream of KRAS. The mTOR inhibitor Temsirolimus had a higher predicted IC50, meaning a decreased sensitivity, in the high-m5C-score group (Figure 7A). On the contrary, inhibition of AKT by A.443654, of MEK1/2 by PD.0325901 and RDEA119, and of PI3Kβ by AZD6482 was predicted to be more efficient in the high-m5C-score group in agreement with the high gene expression of AKT1-3, MEK1 and PIK3CB (Figures 7B–D). This indicated that tumors with high m5C-score, which exhibit higher KRAS mutation and expression (see Figures 5A,B), might be eligible for targeted therapy with inhibitors of AKT, MEK or PI3K, but not mTOR.

PDAC is characterized by frequent overexpression of ERBB receptors upstream of KRAS, notably EGFR and HER2 (ERBB2). Targeted therapy against EGFR or HER2 has yielded contrasted results in clinical trials, but combined targeting showed a synergistic effect in preclinical models (Maron et al., 2013; Liu et al., 2021). The expression of both EGFR and ERBB2 was increased in the high-m5C-score group compared to the low-m5C-score group (Figure 7E). In the high-m5C-score group, the predicted sensitivity was reduced for the EGFR inhibitor Gefitinib but increased for Lapatinib, an inhibitor of both EGFR and HER2, indicating that a combined inhibition might be effective in tumors with a high m5C-score (Figure 7E).

A subset of sporadic PDAC is characterized by DNA repair efficiency due to mutations in the homologous recombination (HR) pathway genes BRCA1/2 and/or PALB2. The inhibition of PARP, a downstream HR gene, has proven to be efficient for the treatment of BRCA1/2-mutated PDAC. The expression of BRCA1/2 and PALB2 was reduced and the predicted sensitivity to the PARP-inhibitor ABT.888 was accordingly increased in tumors with a low m5C-score compared to the high-m5C-score group (Figure 7F).

Overall, tumors with a high m5C-score were predicted to be more sensitive to AKT, MEK and PI3K inhibitors, as well as combined EGFR/HER2. In contrast, tumors with low m5C-score might be more sensitive to PARP inhibition.

Subsequently, we determined the m5C-score in different PDAC cell lines. We compared the m5C-score in 17 different pancreatic cell lines included in the Moffit classification study (Moffitt et al., 2015). The Panc1005 cell line showed highest m5C-score, followed by CAPAN1, while PANC1 showed the lowest m5C-score (Figure 7H and Supplementary Table S8-7). In addition, the sequencing data from four pancreatic cancer cell lines (CAPAN1, PANC1, MIAPACA2 and CFPAC1) was acquired from GSE165949 (Alfarano et al., 2022). CAPAN1 showed the highest m5C-score, the PANC1 showed the lowest m5C-score, while MIAPACA2 cells had an almost negative m5C-score, confirming the results above (Figure 7G; Supplementary Table S8-8). These results might serve as basis for further studies using cell lines to compare the biological effects of high and low m5C-score.

### An m5C-score is associated with immune exclusion and immune resistance

**FIGURE 8:**
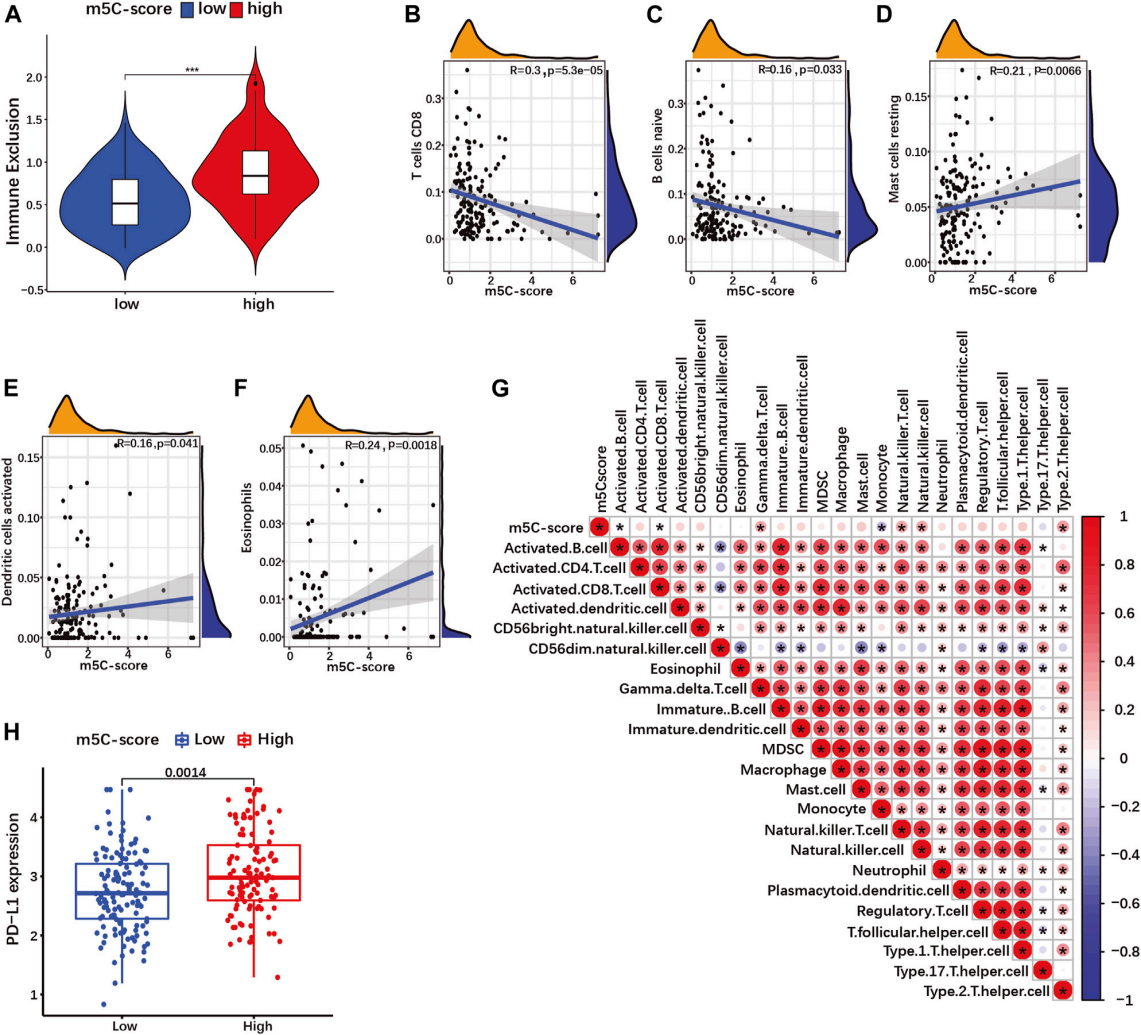
The m5C-score correlates with immune exclusion, low cytotoxic immune cells infiltration and PD-L1 expression. **(A)** Immune exclusion score between high- and low-m5C-score groups in the TCGA-PAAD cohort. **(B–F)** Spearman correlation of immune cells infiltration with m5C-score in TCGA-PAAD and GSE57495 cohorts analyzed by CIBORSORT. **(G)** Spearman correlation of immune cells infiltration with m5C-score in TCGA-PAAD and GSE57495 cohorts analyzed by ssGSEA. *p-value<0.05. **(H)** Expression of PD-L1 between high- and low-m5C-score group in the TCGA-PAAD and GSE57495 cohorts.

Recent studies have suggested a role of RNA modification in cell infiltration within the tumor microenvironment (TME) (Xu F. et al., 2021; Zhang M. et al., 2021). Therefore, we investigated the overall abundance of tumor infiltrating immune cells (TIICs) in the TCGA-PAAD cohort. Immune exclusion was significantly higher in tumors with high m5C-score compared to the low-m5C-score group (Figure 8A; Supplementary Table S12). Next, we evaluated the relative abundance of 25 different types of TIICs in every PDAC sample in our cohort (Supplementary Table S13), and determined the correlation between m5C-score and the various TIICs. The m5C-score correlated negatively with the presence of CD8^+^ T cells (Figure 8B) and naive B cells (Figure 8C), and was positively with to the presence of resting mast cells (Figure 8D), activated dendritic cells (Figure 8E) and eosinophils (Figure 8F). Then, ssGSEA was used to calculate the relative enrichment of immune cells in tumor microenvironment (TME) of PDAC patients in our cohort (Supplementary Table S14). Here, the m5C-score correlated positively with the infiltration of gamma delta T cells, natural killer T cells, natural killer cells and type 2 T helper (Th2) cells, and negatively with the infiltration of activated B cells, activated CD8^+^ T cells, and monocytes (Figure 8G). Taken together, these results showed that a high m5C-score was associated with more frequent immune exclusion, a type 2 immune response (mast cells, eosinophils, Th2 T cells), and a low cytotoxic T cell infiltration and activity (CD8^+^ T cells), all indicative of an immune-deprived microenvironment with low anti-tumorigenic activity.

Overexpression by tumor cells of immune checkpoint ligands such as the programmed death ligand 1 (PDL1) represents a mechanism of immune escape, but also a therapy target. In our cohort, PD-L1 expression was significantly more expressed in high-m5C-score tumors compared to low-m5C-score tumors (Figure 8H). Altogether, tumors with a high m5C-score displayed characteristics of immunologically “cold” cancers and immune evasion.

### YBX1 expression correlates with m5C-score, target drug IC50, immune exclusion and immune infiltration.

Since YBX1 had the best predictive power for survival among the m5C-regulators, albeit lower than the m5C-score (see Figure 4E), we analyzed the relationship between YBX1 expression level and the m5C-score, predicted targeted drug response and immune infiltration. The m5C-score was significantly higher in the high-YBX1 expression level group compared to the low-YBX1 expression group (Supplementary Figure S6A). Next, we estimated the IC50 of target drugs between high and low YBX1 expression groups (for drugs significantly related to the m5C-score). Only two target drugs showed a significant difference. Similar to the m5C-score, high YBX1 expression was associated with a higher sensitivity to the PI3Kβ inhibitor AZD6482, and a lower sensitivity to the EGFR inhibitor Gefitinib (Supplementary Figures S6B,C). Contrary to the m5C-score, YBX1 was not associated with the response to inhibitors of AKT, MEK, combined HER1/HER2 or PARP. Then, we analyzed the correlation of YBX1 with immune exclusion and immune infiltration. Tumors with high YBX1 expression had more immune exclusion compared to the low YBX1 expression group (Supplementary Figure S6D). CIBERSORT analyses showed that YBX1 expression correlated negatively with mast cells, and positively with CD4^+^ memory T cells in a significant manner. Using ssGSEA analyses, we found a positive correlation between YBX1 expression and many kinds of immune cells such as activated B cell, activated CD8^+^ T cell, activated dendritic cell, natural killer cell and type and type 1 and 2 T helper cell (Supplementary Figure S6G). PD-L1 expression was significantly up-regulated in the high YBX1 expression group compared to the low YBX1 expression group (Supplementary Figure S6H). Overall, these results showed that the YBX1 expression correlates with the m5C-score, but the m5C-score was a better predictor for prognosis and drug sensitivity. Contrary to the m5C-score, YBX1 expression was associated with an active anti-tumor immune response. However, both factors were equally associated with PDL-1 expression and immune exclusion.

## Discussion

An increasing body of studies suggests that m5C modification regulators are involved in tumorigenesis, tumor progression, and anti-tumor immune response in multiple malignancies including breast, ovarian, cervical or prostate cancers (Esteve-Puig et al., 2020; Nombela et al., 2021). In the present study, we aimed to comprehensively investigate the predictive value of m5C regulation in PDAC. Overall, we observed a low level of CNVs or SNVs for m5C-regulator genes in PDAC samples and the presence of genomic alterations did not correlate with patient survival, indicating that mutations of m5C-regulator genes were rather passenger than driver mutations. These findings were in accordance with a recent study by Yu et al. (2021), who uncovered a small number of m5C-regulator gene mutations in PDAC, that were without predictive statistical significance. In a pan-cancer study across 33 tumor types, He et al. (2021) also reported low mutation frequency among m5C-regulators. At the RNA level, we found that most of the m5C-regulators were differentially expressed between normal and tumor tissues, or between different tumor samples, confirming that m5C-regulator gene expression is frequently dysregulated in tumors (He et al., 2021). The prognostic value of m5C-regulator gene expression has been assessed in several other tumor entities. Several m5C-regulators are associated with overall survival prognosis in cutaneous melanoma (DNMT2, NSUN1, NSUN3, NSUN6 and YBX1). In particular, low NSUN6 expression was shown to correlate with melanoma progression (Huang et al., 2021a). On the contrary, in lung squamous carcinoma, while most of the m5C-regulators showed significantly different expression between tumor and normal samples, only NSUN3 could significantly predict prognosis (and NSUN4 almost significantly) (Pan et al., 2021). NSUN6 expression could predict prognosis in both breast and colorectal cancer. However, NSUN6 expression was associated with a poor prognosis in breast cancer, and with a more favorable prognosis in colorectal cancer (Huang et al., 2021b; Fang et al., 2022). In the present study, NSUN6 expression was associated with a longer survival. Out of the seven genes whose expression correlated with survival, only YBX1, an m5C-binding protein, was significantly able to predict prognosis in PDAC. Hence, it appears that while the expression of m5C-regulators is often associated with prognosis in several tumor types, which specific genes are involved and the prognostic value of each single gene vary from entity to entity.

Since only one single m5C-regulator (YBX1) could predict prognosis in our study but a high level of correlation was found between the expression of the different m5C-regulators, we investigated whether m5C-regulator-associated expression profiles were related to prognosis in PDAC. Patient clustering according to m5C-regulator expression did not correlate with survival in our PDAC cohort. However, we could identify up to 100 genes differentially regulated between the three m5C-clusters, that showed prognostic value. Based on these prognostic DEGs, we identified three m5C-prognostic-gene clusters, which could stratify PDAC patients. Notably, the m5C-prognostic-gene cluster 2 was associated with high prognostic DEG expression, and poor survival. In addition, high expression of m5C-regulators was observed in the m5C-prognostic-gene cluster 2, confirming the validity of the clustering. These data are supported by the fact that the expression of m5C-related long noncoding RNAs (lncRNAs) were found to correlate with prognosis in PDAC (Yuan et al., 2021; Liu et al., 2022). They also indicated that, while m5C-regulators themselves could not predict prognosis for PDAC patients, the profound pro-tumorigenic transcriptional changes induced by m5C-dysregulation could be used to stratify patients and predict disease outcome. This effect might be attributed to the redundancy of function of some m5C-writers, and/or to compensatory mechanisms.

In order to predict the prognosis of patients with PDAC based on the expression of m5C-related prognostic DEG expression, we constructed a risk score (m5C-score) including four prognostic DEGs and classified patients into high- and low-risk groups. Overall, the m5C-score was well suitable for patient stratification. It significantly correlated with survival and successfully predicted PDAC patient prognosis in our cohort, as well as two additional independent cohorts. A similar approach was used by Li et al. (2021) in papillary thyroid carcinoma based on DEGs between three m5C-regulator clusters. In agreement with our results, a high m5C-score correlated with a lower survival rate. In our study, the m5C-score could independently predict prognosis in PDAC patients. It represented a stronger prediction factor than tumor grade, stage or classical PDAC markers such as CEA, MUC16, MUC1, CA199, KRT19 and NSE, and also performed better than any m5C-score gene taken individually. The comparison of high- and low-m5C-score groups offered a further validation by revealing that a high m5C-score was associated with a more aggressive tumor phenotype. For instance, tumors with high-m5C-score displayed more mutations, notably for KRAS, TP53, SMAD4 and CDKN2A, as well as a higher KRAS expression. In addition, cancer pathway expression including the Ras, p53, MAPK, ERBB and TGF-beta PI3K-AKT pathways was enriched in the high-m5C-score group. Furthermore, there was an association between high m5C-score and the prognostically unfavorable squamous/basal molecular pathway using all three PDAC classifications from Bailey, Collisson and Moffitt (Collisson et al., 2011; Moffitt et al., 2015; Bailey et al., 2016). Interestingly, therapy response prediction showed that the high m5C-score group was more likely to respond to therapy targeted at the PI3K-AKT, MEK, ERBB pathways. This was well in agreement with the elevated activation of these pathways in tumors with high m5C-score. On the contrary, tumors with a high m5C-score were predicted to have a lower sensitivity to PARP inhibition than low-m5C-score tumors. This might be explained by the fact that m5C-methylation of mRNA is involved in DNA damage repair, notably by promoting homologous recombination (HR). Chen et al. have recently shown that the RNA methyltransferase TRDMT1 is recruited to DNA damage sites to promote m5C-methylation. Loss of TRDMT1 compromised HR, increased cellular sensitivity to DNA double-strand breaks and confered sensitivity to PARP inhibitors *in vitro* and *in vivo* (Chen et al., 2020). Finally, a high m5C-score correlated with more frequent immune exclusion, immune exhaustion (low activated cytotoxic T-cells), and immune evasion (high PDL-1 expression). These findings indicated that m5C phenotype-associated patterns also affect the TME, and that the m5C-score might be used as a marker for immunologically “cold” cancers and immune evasion. This is in line with the fact that m6A-regulators have been reported to correlate with anti-tumor immunity in PDAC, and have been proposed to regulate the immune microenvironment (Xu F. et al., 2021). Notably, a high m6A-risk signature associated with poor prognosis was found to correlate with lower naive B cells and CD8^+^ T cells infiltration, similarly to what we observed for the high m5C-score (Xu F. et al., 2021). More generally, RNA methylation has been shown to inhibit RNA recognition by Toll-like receptors or dendritic cells, to regulate T-cell differentiation and expression of immune factors such as IL-17, or to modulate macrophages polarization (Zhang M. et al., 2021).

When comparing YBX1 expression with the m5C-score, we found that the m5C-score was a better predictor of prognosis and drug sensitivity. YBX1 codes for the Y- box binding protein-1, a multifunctional oncoprotein regulating cell proliferation, survival, drug resistance in cancer (Kuwano et al., 2019). YBX1 can act as transcription factor, but is also involved in DNA repair and RNA splicing. In agreement with our findings, overexpression of YBX1 has been reported in PDAC, where it regulates cell-cycle progression and proliferation through the expression of cell-cycle-related cyclins and GSK3B (Liu et al., 2020). However, YBX1 is not specific to m5C, can bind several other modifications or be activated by oncogenic pathways (Ban et al., 2020; Alkrekshi et al., 2021; Feng et al., 2021). In conclusion, even if YBX1 represents a good prognostic marker, it is not specific of m5C-methylation and could therefore complement but not substitute the m5C-score.

Taken together, our results demonstrated that the m5C-score represents a robust prognostic tool for patients with PDAC, which correlates with molecular subtype as well as an immune-deprived and immune-resistant tumor microenvironment.

## Data Availability

Publicly available datasets were analyzed in this study. This data can be found here: Gene Expression-Omnibus (GEO) (https://www.ncbi.nlm.nih.gov/geo/): GSE21501; GSE71729; GSE17891; the Cancer Genome Atlas (TCGA) (https://portal.gdc.cancer.gov/): TCGA-PAAD; https://www.nature.com/articles/nature16965#Sec11.
