# Supplementary material for: An m5C methylation regulator-associated signature predicts prognosis and therapy response in pancreatic cancer
Source: Front Cell Dev Biol. 2022 Aug 19;10:975684. doi: 10.3389/fcell.2022.975684 (PMC9437259; doi:10.3389/fcell.2022.975684)
Supplement: Supplementary file 6 [file DataSheet1.PDF]

Supplementary Material

Supplementary Figures 1 to 6:

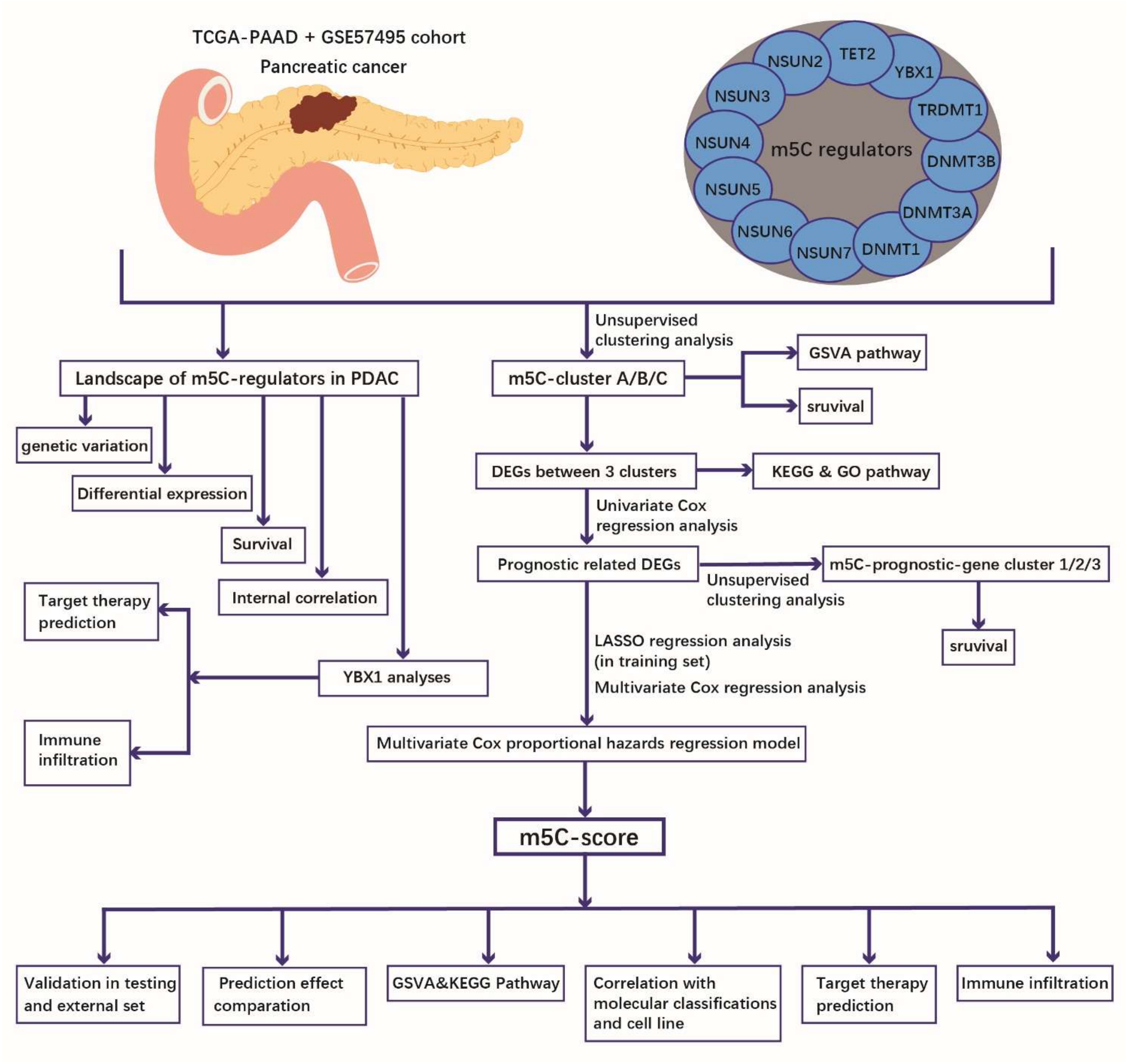

Supplementary Figure 1. Workflow of analysis.

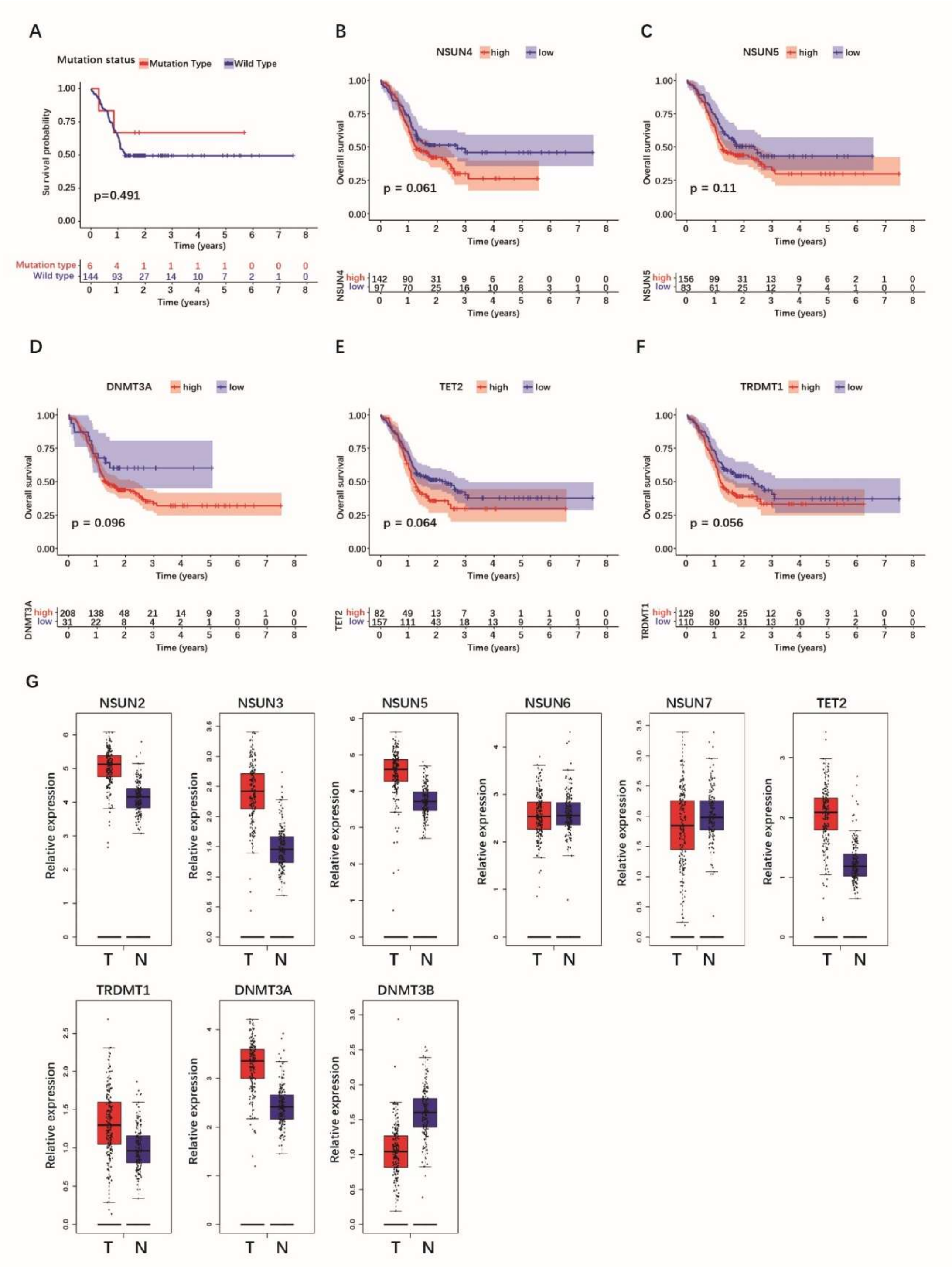

Supplementary Figure 2. K-M curves of patient overall survival (OS) analyzed by log-rank test for (**A**) PDACs with mutation(s) of m5C-regulator-genes versus wild-types in the TCGA-PAAD cohort and (**B-F**) PDACs with high-versus low-expression of m5C-regulator genes in the TCGA-PAAD and GSE57495 cohorts. (**G**) Differential expression of m5C-methylation regulators between PDAC and normal pancreas tissue in the TCGA-PAAD and GTEx cohorts. Tumor, red; Normal, blue.

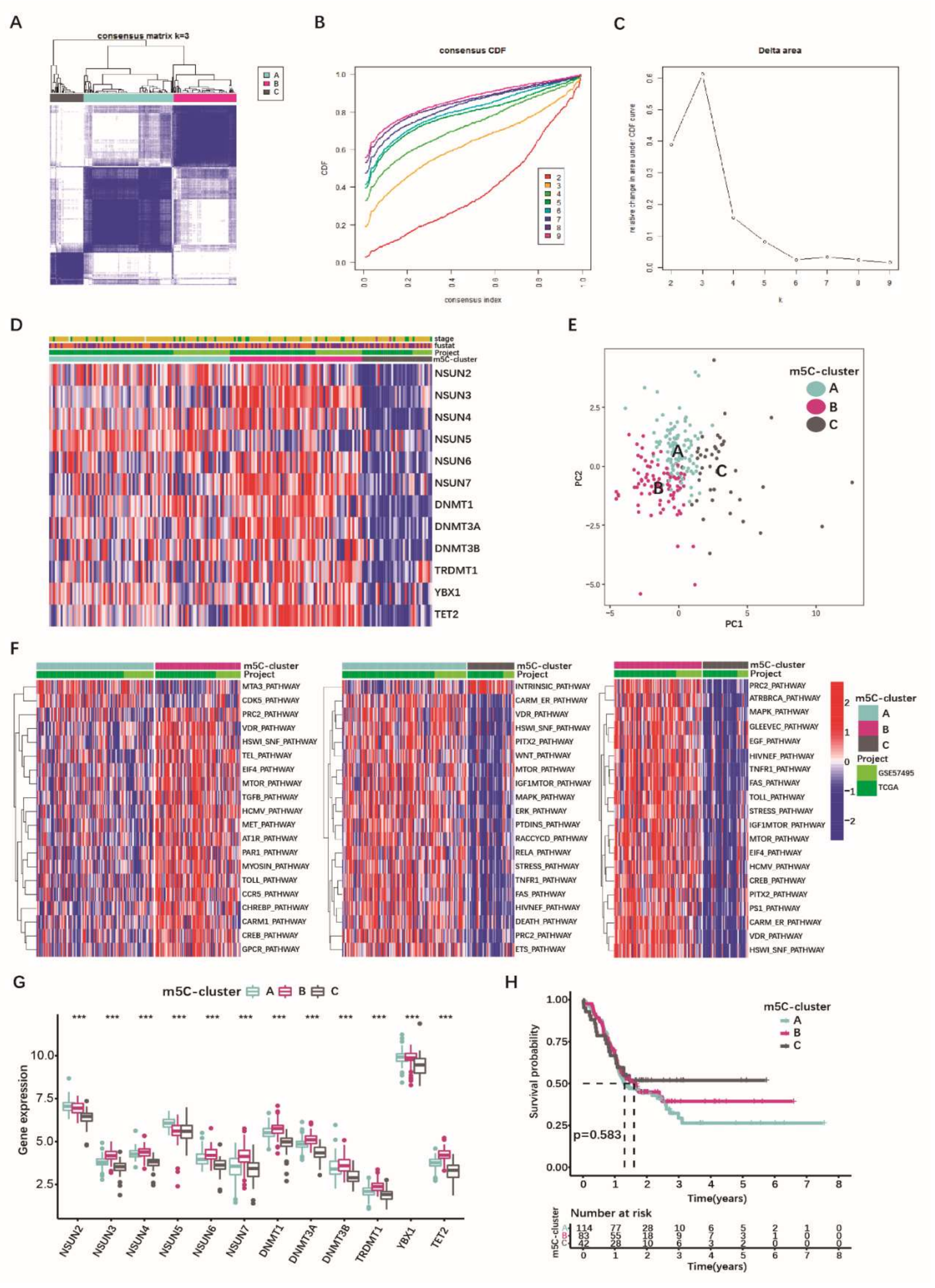

Supplementary Figure 3. m5C-regulator gene expression clusters (m5C-clusters) are associated with RNA processing and cancer-related pathways, but not survival. (A-C) Consensus clustering analysis of the TCGA-PAAD and GSE57495 cohorts based on the expression of m5C-regulator genes using the optimal k value=3. (D) Heatmap showing the expression of 12 m5C-regulator genes in the 3 m5C-clusters with annotations of survival status, stage and project. Columns represent patients and rows represent m5C-regulator genes. (E) Principal components analysis (PCA) showing patients of the m5C-clusters A, B and C. (F) Gene set variation analysis (GSVA) showing different enriched pathways between m5C-clusters A, B and C. (G) The expression of individual m5C-regulator genes in the different m5C-clusters; *p<0.05; **p<0.01; ***p<0.001. (H) K-M curves showing the different OS between m5C-clusters A, B and C analyzed by log-rank test.

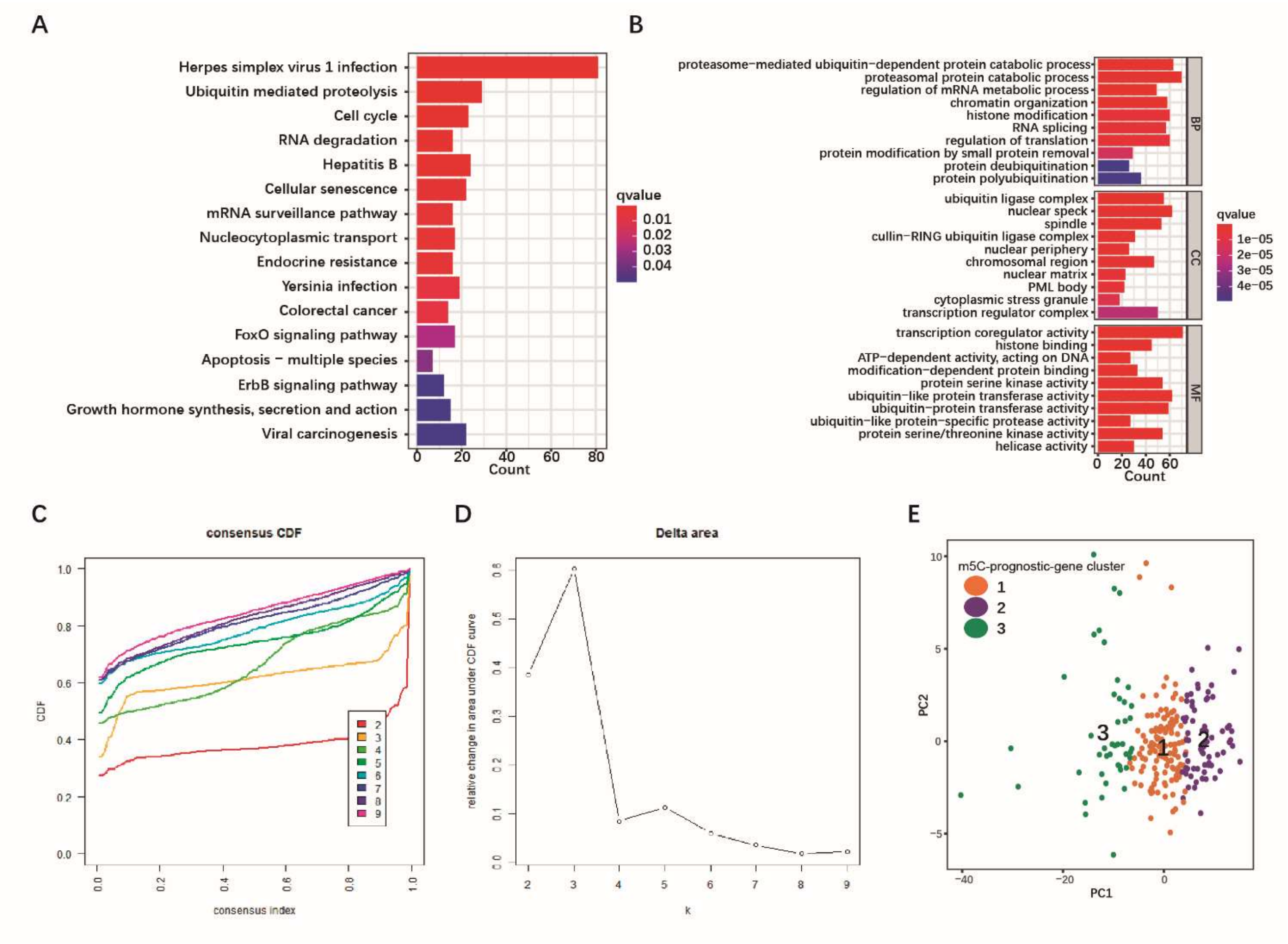

Supplementary Figure 4. Identification of m5C-prognostic-gene clusters. (**A-B**) Kyoto Encyclopedia of Genes and Genomes (KEGG, A) and Gene Ontology (GO, B) annotation enriched pathways based on DEGs between m5C-clusters A, B and C. (**C-D**) Unsupervised consensus clustering analysis based on prognostic m5C-related DEGs showing the optimal k value of 3. (**E**) Principal components analysis (PCA) of PDAC patients of the TCGA-PAAD and GSE57495 cohorts in the m5C-prognostic-gene clusters 1, 2 and 3.

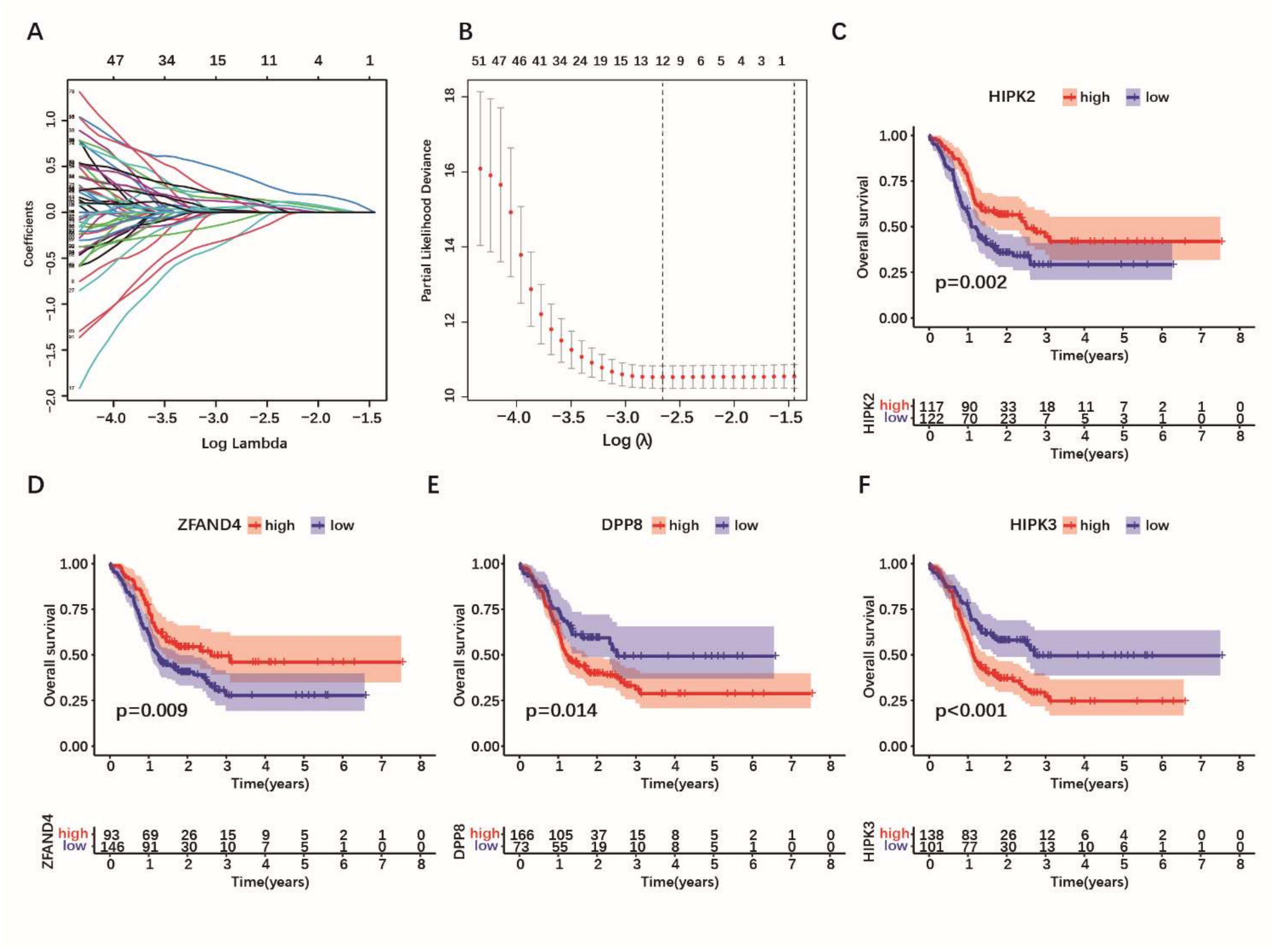

Supplementary Figure 5. Construction of a four-gene scoring signature (m5C-score) prognostic model. (**A-B**) The coefficients of genes are confirmed by LASSO algorithm with minimal lambda. (**C-F**) K-M curves showing significant differences of OS between high- and low-expression of each individual m5C-score genes analyzed by log-rank test in the TCGA-PAAD and GSE57495 cohorts.

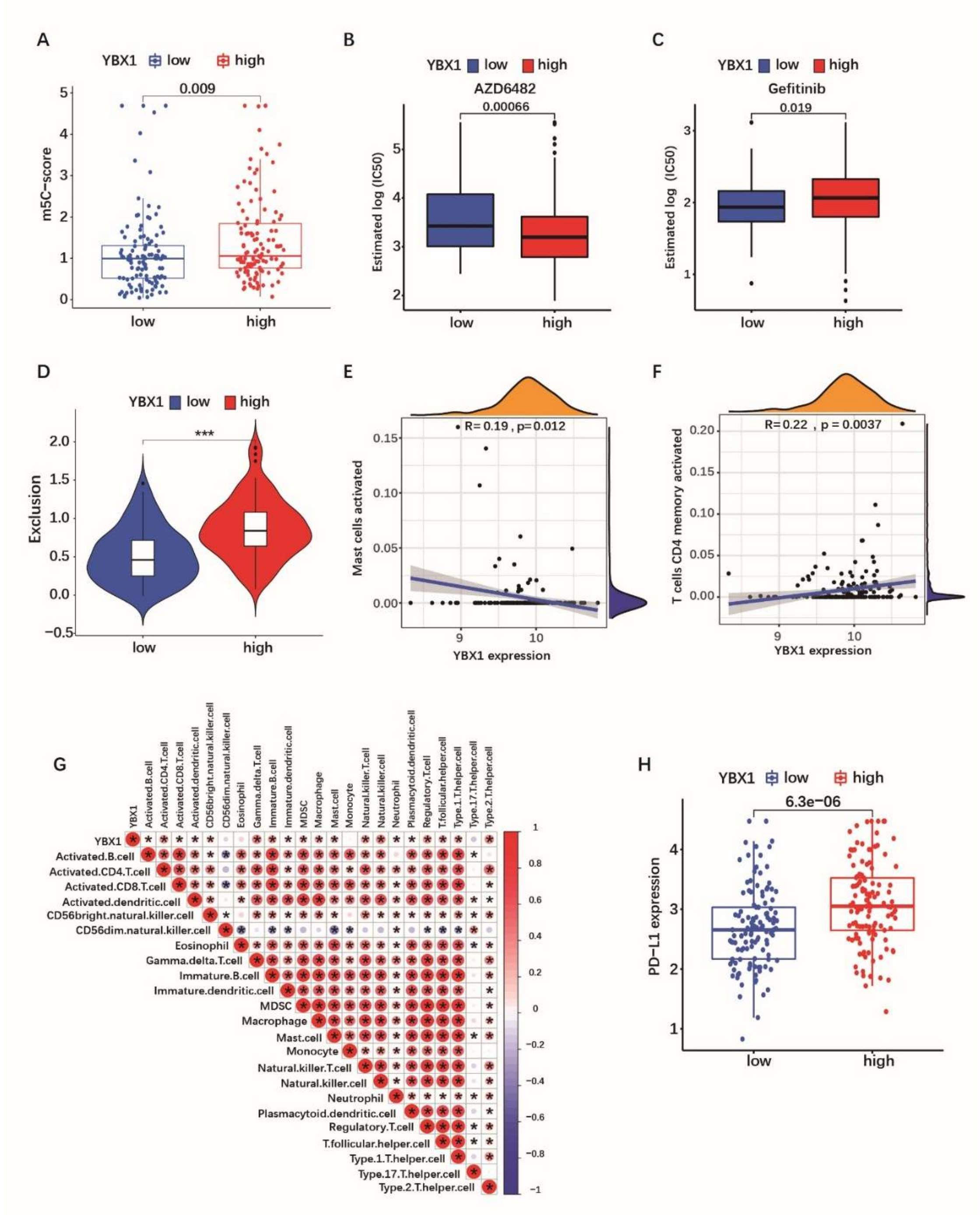

Supplementary Figure 6. YBX1 expression correlates with m5C-score, target drug IC50, immune exclusion and immune infiltration. (**A**) Comparison of m5C-score value between high- and low-YBX1 expression groups in TCGA-PAAD and GSE57495 cohorts. (**B-C**) Target drug IC50 prediction for AZD6482 and Gefitinib in high- and low-YBX1 expression group from the TCGA-PAAD and GSE57495 cohorts. (**D**) Immune exclusion score between high- and low-YBX1 expression groups in the TCGA-PAAD cohort. (**E-F**) Spearman correlation of immune cells infiltration with YBX1 expression level in the TCGA-PAAD and GSE57495 cohorts analyzed by CIBORSORT. (**G**) Spearman correlation of immune cells infiltration with YBX1 expression level in the TCGA-PAAD and GSE57495 cohorts analyzed by ssGSEA. (**H**) Expression of PD-L1 between high- and low-YBX1 expression groups in the TCGA-PAAD and GSE57495 cohorts. * p-value<0.05, ** p-value<0.01, *** p-value<0.001.
